# Comments on Dermatologists’ TikTok Videos on Atopic Dermatitis: Content Analysis

**DOI:** 10.2196/90649

**Published:** 2026-07-16

**Authors:** Sahithi Gangavarapu, Tazeena Khan, Morgan McCarthy, Molly Hales, Stephanie M Rangel

**Affiliations:** 1 Department of Dermatology, Feinberg School of Medicine, Northwestern University, 676 N. St. Clair Street, Suite 1600, Chicago, IL, 60611, United States, (312) 503-5942; 2 College of Medicine, University of Illinois Chicago, Chicago, IL, United States; 3 Chicago College of Osteopathic Medicine, Midwestern University, Downers Grove, IL, United States; 4 Department of Dermatology, UChicago Medicine, University of Chicago, Chicago, IL, United States

**Keywords:** atopic dermatitis, eczema, TikTok, social media, dermatology, peer support, online community, online engagement

## Abstract

**Background:**

TikTok is one of the fastest-growing social media platforms in the world. It has become an important space for sharing information on a wide range of topics, including medical conditions such as atopic dermatitis (AD). Advice on skin conditions has become popular on TikTok, and most previous research in this area focuses on the credibility of the information being shared. However, little research has focused specifically on physician-created videos and their audience engagement and interaction.

**Objective:**

Our study aimed to (1) characterize the audience’s online response to board-certified physicians’ TikTok content related to AD according to established protocols and (2) better understand the interactions that happen among members of the audience in the comment section of this content.

**Methods:**

In December 2023, searches were conducted for the terms “atopic dermatitis” and “eczema” on 3 unique TikTok accounts to identify videos created about AD by board-certified dermatologists. A total of 28 final videos were analyzed and classified into the following categories: (1) explanation of disease, (2) recommendation, (3) debunking misinformation, and (4) informal or anecdotal. The top 50 original comments on each of the 28 videos were collected and classified into one of the following categories: (1) “positive personal experience,” (2) “negative personal experience,” (3) “neutral personal experience,” (4) “requesting advice,” (5) “learning,” (6) “appreciative reaction,” (7) “critical reaction,” (8) “giving advice,” (9) “humor,” (10) “tagging another user,” and (11) “off-topic.” Replies to comments were also analyzed and grouped into similar categories.

**Results:**

Video category did not have a significant impact on engagement rate (*P*>.99). Across all video categories, comments that involved personal experiences or sharing information made up a larger percentage than those that were critical or off-topic (*P*<.001). Of the comments related to personal experience, the percentage of negative personal experience comments was significantly higher than that of positive personal experience comments (*P*=.001). Among replies to comments, “recommendation” and “emotional support” replies were significantly more common than other types of replies (*P*<.001).

**Conclusions:**

Our study suggests that videos created by dermatologists on TikTok are generally well received regardless of video style or category. The comment sections appear to provide transient supportive environments where users connect over shared challenges and exchange personal experiences and recommendations. There is a gap in dermatologist-produced TikTok content involving darker skin tones.

## Introduction

### Background

Social media platforms have become increasingly popular forums for people who share similar experiences to connect. TikTok has gained traction since its creation in 2016 and has become one of the most popular platforms used by people to share their stories, request advice, and connect with others [1]. Medical content, including videos that discuss symptoms, diagnoses, and treatment of various conditions, is widespread on TikTok, including content pertaining to skin health and diseases.

### Atopic Dermatitis on Social Media

Atopic dermatitis (AD) is a common chronic skin condition with a strong patient presence in social media communities on various platforms, where patients often post about their experiences and concerns related to the disease [2]. AD affects 15% to 30% of children and 2% to 10% of adults in industrialized countries [3,4]. Due to its itchy, painful, and visible nature, AD can often impact self-image and quality of life. Patients with AD have reported lower satisfaction with life scores and lower mental and physical health scores [5]. Previous studies have found that online posts from patients with dermatoses, including AD, often discuss issues such as self-image, self-confidence, and therapeutic options [6].

There are many systemic barriers to care that patients with AD may experience, including financial and geographic barriers as well as patient knowledge and beliefs [7]. In light of these potential access issues, the public nature of videos makes TikTok an easy and accessible way for patients to find information related to their disease. However, misinformation can reach patients just as easily. Studies have found that TikTok content produced by board-certified physicians is more accurate and reliable than content produced by others [8,9], but they do not produce much of the content that reaches patients. One study found that most skin care–related TikTok content was produced by laypeople, and of the most liked and most viewed skin care posts, only 2.5% were produced by board-certified dermatologists [10].

By combining accessibility and accuracy, videos produced by board-certified physicians may be a helpful avenue for patients to obtain accurate information regarding AD. Understanding how people respond to these videos by analyzing the comments they leave may be helpful to determine the utility of such content and assess whether patients are able to find supportive communities on TikTok.

### Patient Experience With Illness-Related Social Media Content

Prior studies have analyzed illness-related content on other diseases on social media to better understand its purpose and utility for patients. Many of these studies have found that most illness-related content is produced by patients or loved ones, and producing content on social media can serve as an outlet for these individuals to discuss their experiences with a disease or treatment [11].

Some studies have attempted to characterize patients’ perceptions of such illness-related content and have found mixed reactions. Concern about misinformation has been cited as a primary reason for disliking health-related social media content, whereas observing empathy and shared experiences has been reported as a primary benefit [12]. This suggests that while patients have varying responses to illness-related content on social media, many appreciate the feelings of relatability and support that this content can produce.

Prior studies have found that public reactions on social media can be reasonably representative of public opinion gathered via conventional surveys [13]. Additionally, multiple studies have used comments on social media to assess and analyze public audience reactions in the context of illness-related content (eg, videos; [14,15]). Naslund et al [16] found that interactions among commenters on social media conveyed themes of hopefulness, minimizing isolation, peer support, and learning from shared experiences. This suggests that commenters may feel support from the initial video or post itself but also from interactions with other users or the video creator in the comment section.

Our study aimed to (1) characterize the audience’s online response to board-certified physicians’ TikTok content related to AD according to established protocols [14,15,17] and (2) better understand the interactions that happen among members of the audience in the comment section of this content. In doing this, we aimed to understand whether the supportive environments that have been observed as a result of patient-driven illness-related content on social media are still observed with physician-driven content related to AD.

## Methods

### Video Identification

Three unique TikTok accounts were used to search 2 separate terms (“atopic dermatitis” and “eczema”) in December 2023. Two accounts were previously established personal accounts, whereas 1 account was newly created for the purpose of the study in an attempt to mitigate the effects of a personalized algorithm. Using the “Top” video categorization on TikTok, the first 30 videos to appear under each term for each account (for a total of 180 videos) were screened for inclusion. From this initial list of 180 videos, duplicate videos (75/180, 41.7%), videos that were not in English (12/180, 6.7%), and videos that did not appear to be posted by a board-certified physician (57/180, 31.7%) were excluded, resulting in 36 videos. To confirm board certification status, we did a preliminary search of the creator’s publicly available full name to find an associated hospital, university, or clinic. On the basis of the location of their associated institution, we cross-checked their certification status with national online databases. This resulted in the use of the American Board of Medical Specialties (United States), General Medical Council (United Kingdom), or Royal College of Physicians and Surgeons of Canada (Canada) databases. Of the remaining 36 videos, 7 were further excluded through this process, and 1 additional video was excluded as the content was not related to AD, leading to 28 final videos to be analyzed. All analyzed videos were posted to the social media platform between 2020 and 2023.

### Categorization

#### Thematic Approach

To analyze these qualitative data, a thematic analysis method was used. An inductive approach was used to identify themes in the content by following the guidelines by Braun and Clarke [17]. After collecting the final videos that met the inclusion criteria, 3 separate reviewers (TK, SG, and MM) watched all videos to familiarize themselves with the data and perform initial coding. Next, after discussion among the reviewers, 4 categories reflecting video themes were generated. A rubric was created with category definitions and examples to aid in the categorization and analysis process. A similar methodology was followed with the comments for categorization and analysis.

#### Video Categorization

From each video, the following information was collected: creator handle; number of creator followers; date of posting; video duration; and number of views, likes, saves, shares, and total comments. Engagement rate was calculated as the sum of the number of video likes, comments, shares, and saves divided by the number of video views. This formula was derived from engagement rate calculations commonly used in the social media industry and in prior literature [18,19].

Through consensus discussion among 3 raters, each of the 28 videos was classified into one or more of the following categories so that videos could be counted in more than one category if they met the respective inclusion criteria: (1) explanation of disease, (2) recommendation, (3) debunking misinformation, and (4) informal or anecdotal. Videos were also tagged if they (1) used the TikTok “stitch” feature (ie, the video creator adds their content to the end of a different video, typically as a response), (2) were sponsored, (3) used a trending sound vs trending song vs original audio, and (4) were inclusivity focused (ie, if the video focused on presentation of AD in darker skin tones) to consider additional factors that may impact engagement.

#### Comment Categorization

**Figure 1.**
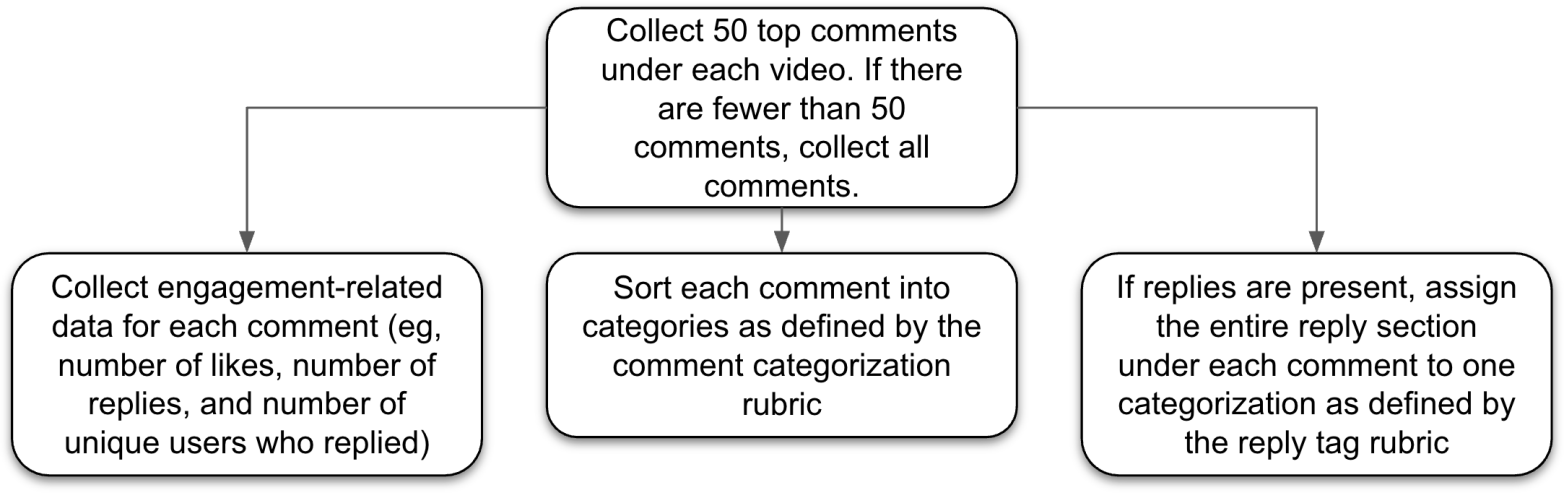
Video comment identification framework for analysis.

For each video, the top 50 original comments (ie, not replies to other comments) were collected. If the video had less than 50 original comments at the time of analysis, all available original comments were collected (Figure 1).

Original comments and secondary comments were divided among 3 individual raters. To confirm interrater reliability, raters first coded a sample of 1.7% (20/1149) of the total comments analyzed and compared their categorizations. We found 80% perfect agreement and 90% agreement among at least 2 raters (Krippendorff α=0.72).

For each original comment, the following information was collected: date, number of likes, whether the creator liked the comment, number of secondary comments (ie, replies to an original comment), number of unique secondary commenters, number of secondary comments from the creator, number of likes on the creator’s secondary comments (if applicable), and number of replies from another public board-certified physician or dermatologist. The original comments were classified into one of the following categories: (1) “positive personal experience,” (2) “negative personal experience,” (3) “neutral personal experience,” (4) “requesting advice,” (5) “learning,” (6) “appreciative reaction,” (7) “critical reaction,” (8) “giving advice,” and (9) “humor.” We also had two categories for unrelated comments: (1) “tagging another user” and (2) “off-topic.” Comments in which users self-identified as patients with AD or relatives or loved ones of patients with AD were tagged.

Additionally, for each original comment with secondary comments, the secondary comment section was scanned and classified into one of the following categories: (1) “recommendation” for discussion focused on requesting and sharing recommendations; (2) “emotional support/shared experience” for discussion focused on relating to, showing support for, or agreeing with the original comment; (3) “argumentative” for discussion that was marked with hostility or nonrespectful disagreement; (4) “educational” for discussion that focused on providing explanations or answers without any recommendations given; and (5) “other” for any secondary comment sections that did not fit into the aforementioned categories.

### Statistical Analysis

**Figure 2.**
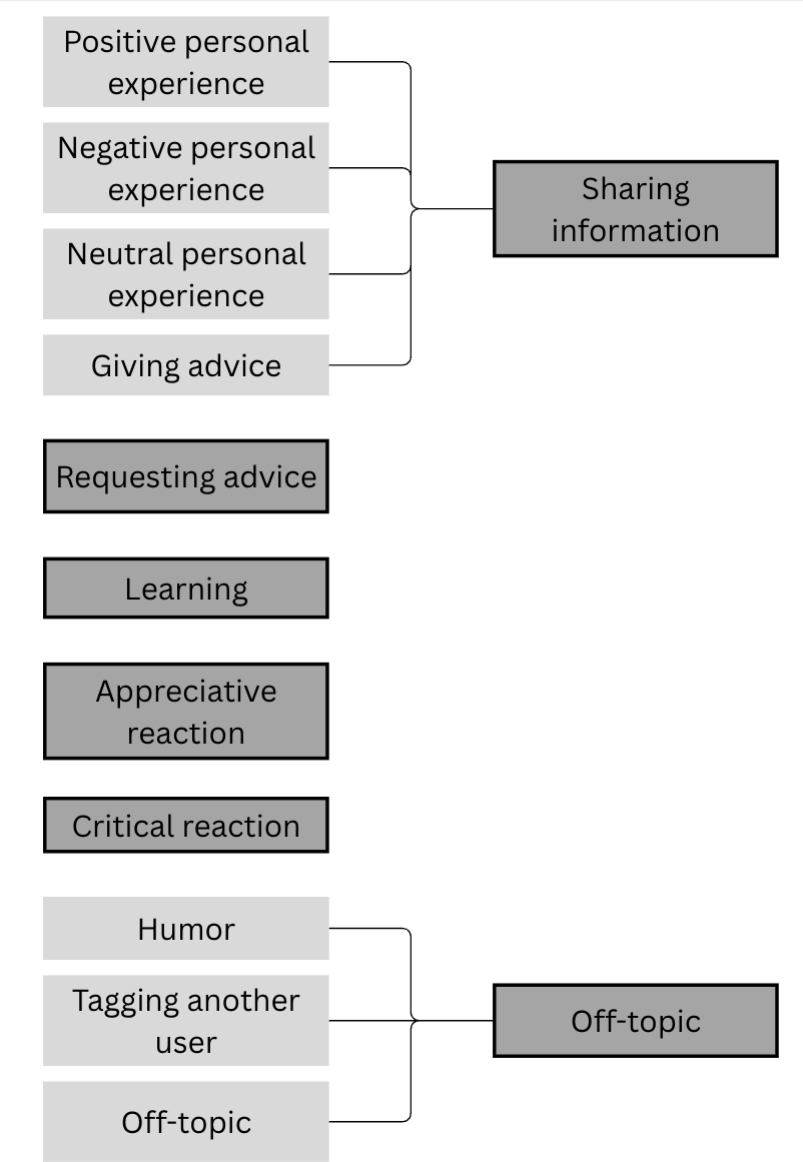
Consolidation of comment categories into broader thematic groups.

All analyses were performed in Prism (version 10.2.3; GraphPad Software), and *P* values of less than .05 were considered statistically significant. Frequency distributions were performed on categorical video, original comment, and secondary comment data separately. Engagement rate analyses were performed using Kruskal-Wallis one-way ANOVAs. For ease of analysis, certain related comment categories were consolidated (Figure 2). “Personal experiences” and “giving advice” were combined into “sharing information.” “Tagging another user,” “off-topic,” and “humor” were combined into a single “off-topic” category.

### Ethical Considerations

This study was exempt from institutional review board approval as there was no involvement with human participants and only publicly available data were used.

## Results

### Video Metrics

**Figure 3.**
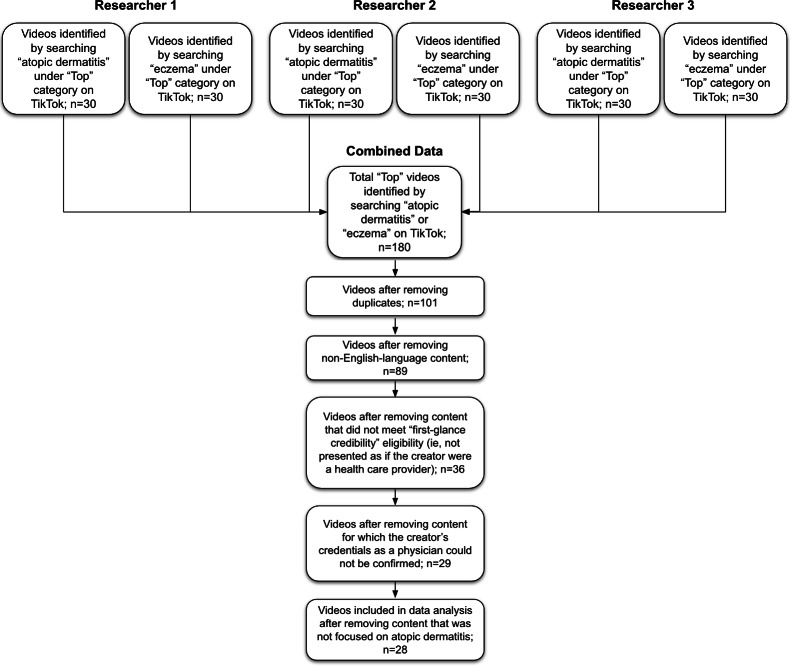
TikTok video identification flowchart for inclusion in the analysis.

The 28 videos analyzed (Multimedia Appendix 1) had a total view count of 98,900,200, with an average of 3,532,150 views per video (SD 9,756,600; range 21,200-51,400,000). Video length ranged from 4 to 89 seconds, with an average length of video of 40.25 seconds. Among the total videos, 7.1% (n=2) were tagged for being inclusivity focused, 7.1% (n=2) were tagged for containing sponsored content, 35.7% (n=10) were tagged for having a “stitched” format, and 53.6% (n=15) were tagged for use of a trending sound or song (Figure 3).

Most videos categorized fell into the “recommendation” category (21/28, 75%), followed by “explanation of disease” (16/28, 57.1%), “debunking misinformation” (5/28, 17.9%), and “informal or anecdotal” (5/28, 17.9%). Most videos fell under 2 categories (Table 1).

**Table 1.** Excerpted transcripts of sample videos for each video category.

Video category	Sample transcript
Recommendation: provides recommendations for people with atopic dermatitis	“One of the first line treatments are topical corticosteroids like triamcinolone which is prescribed frequently. These are safe to use when you follow the directions of your dermatologist, but if you overuse them they can cause thinning of the skin and stretch marks.” [Dr. Portela, 2020] “I was asked to comment on this recent post of diluted bleach baths. Would you let your child swim in a chlorinated swimming pool? Well, diluted bleach baths are really no different, they’re safe and effective at reducing bacteria on infection prone skin and like in children with eczema. Many dermatologists and pediatric dermatologists recommend them routinely. They can be quite helpful.” [Dr. Sheilagh, 2021]
Explanation of disease: includes attempts to explain the presentation, diagnosis, or pathology of atopic dermatitis	“In atopic dermatitis we have an upregulation of this Th2 pathway which causes the physical manifestations and itch. Interestingly, this same pathway is responsible for fighting parasites such as worms.” [Dr. Morris, 2022] “Eczema is also known as atopic dermatitis. When you have this condition you have dry, itchy skin which is really common in kids but can also present for the first time as an adult.” [Dr. Yadav, 2022]
Debunking misinformation: focuses on dispelling myths and misconceptions regarding the disease or treatment	[“Stitched” video of someone saying they found out babies have eczema because they are allergic to dairy so they switched their child’s formula to nutramigin] “This is for educational purposes only, not specific medical advice but I would be careful when considering eliminating a food in a baby’s diet to help treat eczema. The reason being is that many babies who have this—it may not actually be beneficial and could be potentially harmful.” [Dr. Rubin, 2023]
Informal or anecdotal: focuses on the creator’s specific experiences or attempts to be humorous	Text: “When my patient with eczema takes lukewarm showers, trims their nails short, and dunks their body in Vaseline after showers.” Trending audio: “Someone cooked here.” [Dr. Park, 2023]

### Engagement Rate

**Figure 4.**
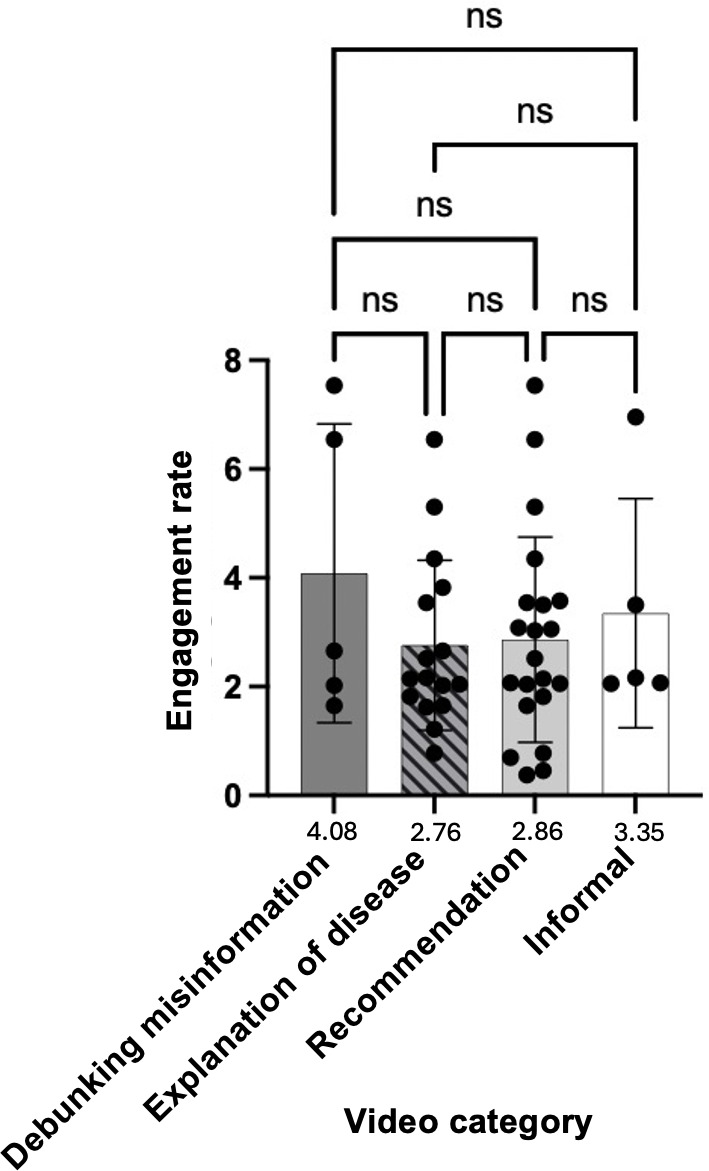
TikTok video engagement rate across all video categories. ns: not significant.

Video engagement rate ranged from 0.38 to 7.54 (Multimedia Appendix 2). Video category did not have a significant impact on engagement rate (Figure 4).

### Comment Categorization Results

The 28 videos analyzed had a total of 30,019 comments, with comment counts on individual videos ranging from 10 to 14,700. For videos with more than 50 comments, the top 50 comments were analyzed, resulting in a total of 1149 comments included in the analysis. The length of time that a video continued to generate top replies varied substantially, and our analysis was limited by the fact that some videos were posted only weeks before the date of analysis. Nevertheless, among the videos we analyzed, the median interval between the earliest and most recent top comment was 268 (IQR 113-477) days.

Of note, 37.2% (427/1149) of the analyzed comments were made by users who self-identified as patients with AD in their comments, and 6.8% (78/1149) were made by users who self-identified as relatives or loved ones of patients with AD (Table 2).

**Figure 5.**
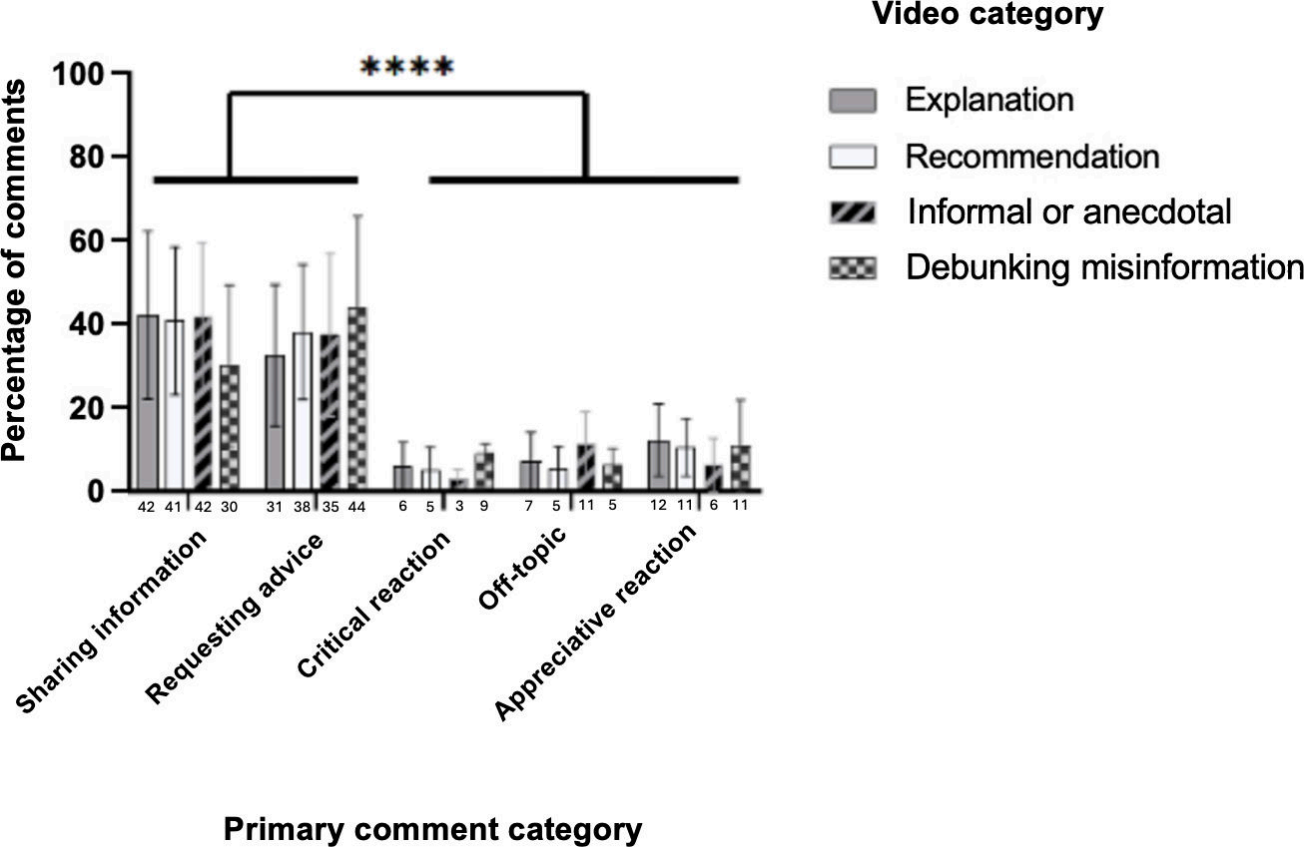
Video comment topics related to sharing information and experiences constituted a greater percentage compared to critical or off-topic comments. ****P<.0001

Across all video categories, “sharing information” and “requesting advice” comments made up a larger percentage than those that were critical, off-topic, or appreciative (*P*<.001; Figure 5). There was no significant difference between the percentage of comments involving sharing information and requesting advice (*P*=.95). Comments that were categorized as “learning” made up only 4.3% (49/1149) of the total.

**Table 2.** Excerpted comments for each comment category.

Comment category	Sample comment
Sharing information	
Positive personal experience: discusses the user’s experience with the disease or product in a positive or praising tone	“This has seriously been a game changer for my daughter. When hers is bad she’ll ask for a bleach bath” [Video 2; comment 2] “4 weeks and my skin is clear! No side effects” [Video 8; comment 24]
Negative personal experience: discusses the user’s experience with the disease or product in a negative or complaining tone	“Ugh it sucks and looks ugly. It’s on my chest, stomach and back mostly... ugh” [Video 14; comment 30]
Neutral personal experience: discusses the user’s experience with the disease or product without praising or complaining	“I notice that I get eczema on my chin /forehead 2 weeks before my period” [Video 12; comment 27]
Giving advice: attempts to provide their own recommendations or resources for other users	“guys use advantan cream it worked so well for me” [Video 17; comment 14]
Requesting advice: asks the creator or other users for more information or specific advice	“How can you differentiate between eczema and allergic reaction” [Video 14; comment 15] “Do all eczema itch?” [Video 16; comment 5]
Learning: expresses that they learned something new	“This explains why my scalp is so clear after being in the pool for a long time!” [Video 2; comment 12] “... THIS HAS A NAME!!!???” [Video 24; comment 22]
Appreciative reaction: shows appreciation to the creator or their overall account, not necessarily for the information presented in the video	“THANK YOU! Pls dont stop sharing!” [Video 25; comment 49] “Thank god I found this video! I have the same thing” [Video 26; comment 23]
Critical reaction: expresses a direct negative reaction or criticizes the creator or their overall account, not necessarily against the information presented in the video	“THIS IS NOT ECZEMA, THIS IS TOPICAL STEROID WITHDRAWAL” [Video 15; comment 42] “Girl you have to explain more because everyone gunna just be taken a bath tub full of bleach” [Video 3; comment 27]
Off-topic	
Humor: did not fit under other categories but was attempting to be humorous	“Honestly, if my arms looked and felt like that, I’d take worms.” [Video 22; comment 37]
Tagging another user: the comment’s only content was tagging someone	@[username]
Off-topic: any other comments, such as spam comments	“First!!” [Video 26; comment 37] “Why do some Americans say ‘egg-zema’ THERES NO G and it’s not an egg” [Video 23; comment 31]

### Personal Experience Comments

**Figure 6.**
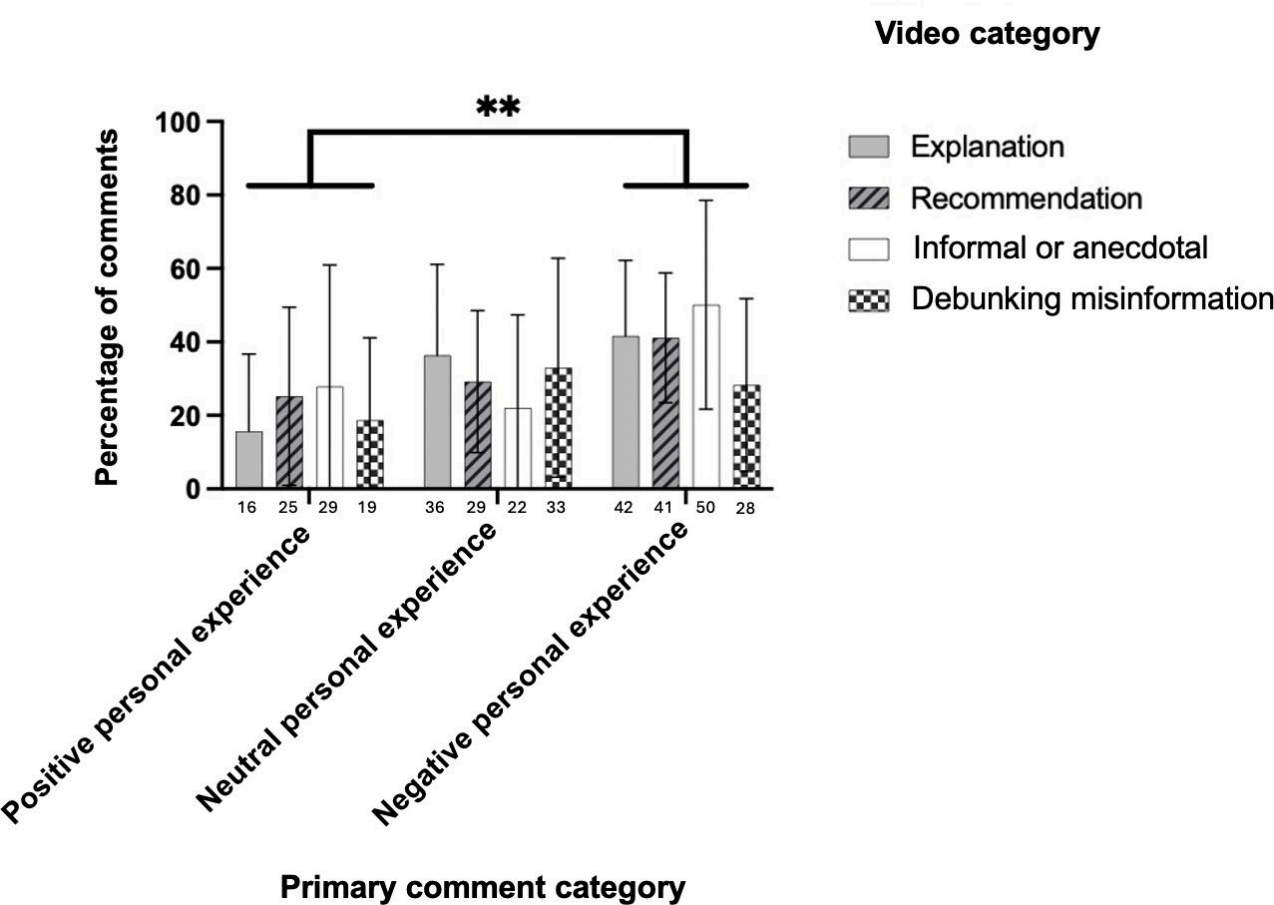
Across all video categories, comments focused on sharing negative experiences with atopic dermatitis vs neutral or positive experiences. **P<.01

Of the comments related to personal experience, the percentage of negative personal experience comments trended higher than that of neutral or positive personal experience comments across all video categories. The percentage of negative personal experience comments was significantly higher than that of positive personal experience comments (*P*=.001; Figure 6); however, there was no significant difference between the percentage of neutral and negative personal experience comments across video categories or between the percentage of positive and neutral personal experience comments.

### Secondary Comment Results

**Figure 7.**
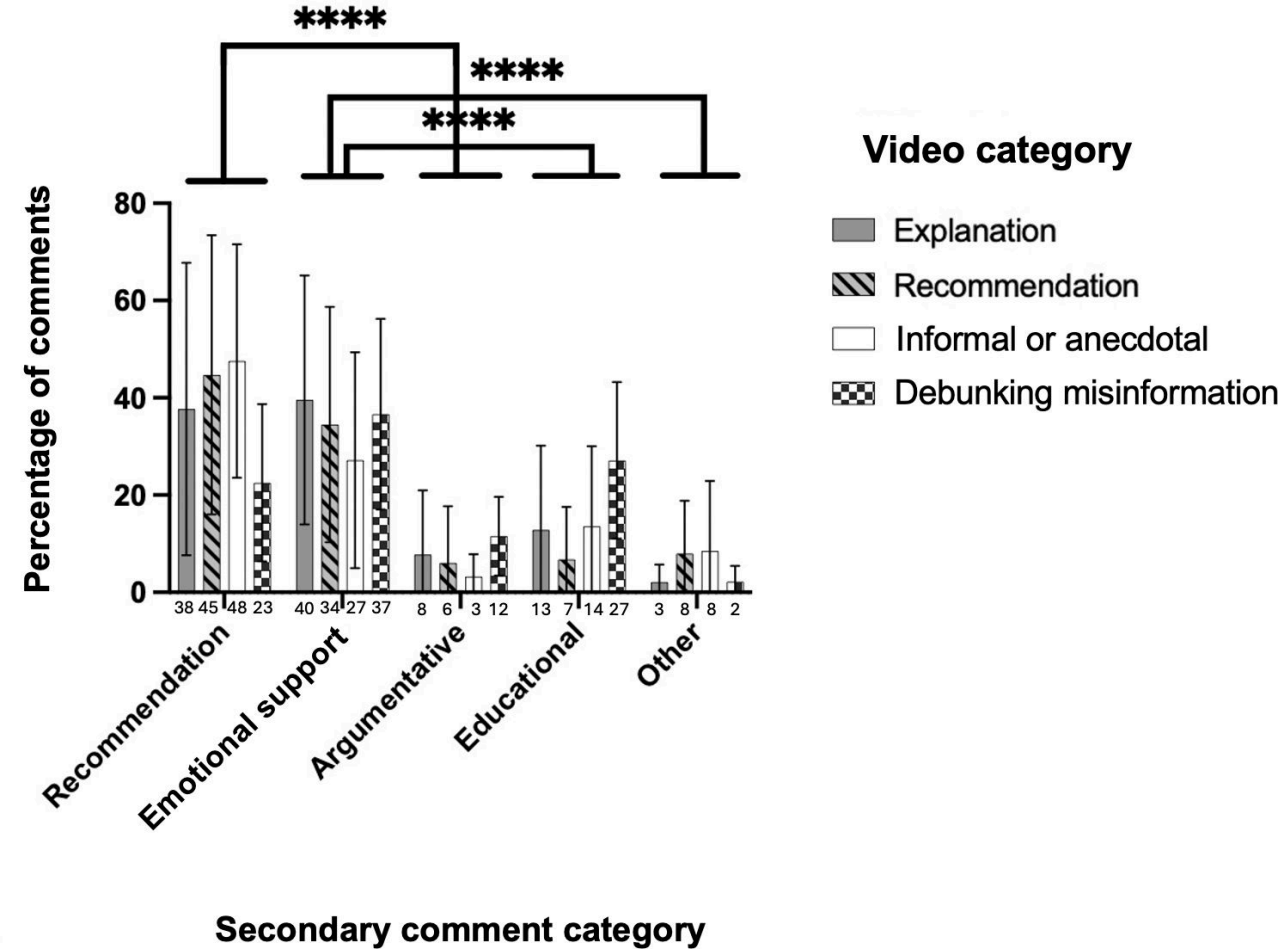
“Recommendation,” “personal experience,” and “emotional support” secondary comments trended higher than argumentative or educational comments. ****P<.0001

Video category did not have a significant impact on the distribution of secondary comment categories. Across video categories, the percentage of “recommendation” secondary comments was significantly higher than the percentage of “argumentative” (*P*<.001), “educational” (*P*<.001), and “other” (*P*<.001) secondary comments (Figure 7). Across video categories, the percentage of “emotional support” secondary comments was also significantly higher than the percentage of “argumentative” (*P*<.001), “educational” (*P*<.001), and “other” (*P*<.001) secondary comments (Figure 7). There was no significant difference between the percentage of “recommendation” and “emotional support” secondary comments (*P*=.96). There were also no significant differences between the percentage of “argumentative,” “educational,” and “other” secondary comments (Figure 7). The number of creator replies did not have a significant impact on the distribution of secondary comment categories (data not shown).

## Discussion

### Principal Results

#### Video Content

Research has shown that most videos related to dermatology on TikTok are created by laypeople rather than board-certified dermatologists [10], and few previous studies have specifically analyzed TikTok content produced by physicians. Among the 28 physician-produced videos we analyzed, format, content, and tone varied widely even within a single category. For instance, in the “recommendation” video category, some videos adopted a formal, explanatory, and detailed approach that was typically longer, whereas others opted for shorter and more casual formats. In the “explanation of disease” category, some videos formally explained the scientific pathogenesis of AD, whereas others offered brief, visual representations of the condition.

Our study found that video category did not affect engagement rate (Figure 4), suggesting that physicians can be versatile with their content and still receive engagement. Only 7.1% (2/28) of the top-appearing videos contained sponsored content, suggesting that sponsorship is not an important component of a successful TikTok video. In total, 28.6% (8/28) of the videos contained a trending sound, whereas 46.4% (13/28) used an original audio, showing that following specific audio formats or trends may not be necessary either. Additionally, the video with the greatest number of views or highest engagement rate was not created by the physician with the greatest number of followers. These findings suggest that physicians can be flexible with the format, content, and length of their videos and produce active engagement regardless of social media experience or an existing large follower base.

We noted that none of the videos analyzed showed practical demonstrations of the techniques being recommended (eg, preparation of bleach bath or application of emollients), instead relying on verbal discussion and occasionally photos. We believe that physician creators could use the video format supported by TikTok and similar platforms to provide such demonstrations, making their recommendations easier for patients to understand.

#### Virtual Supportive Environment

Access to physicians continues to be difficult for many people, with 9.5% of Americans being uninsured and 23% being underinsured in 2023 [20]. Access to dermatologists also varies by location as 88% of US counties have 0 dermatologists [21]. Although these videos are not and do not claim to be a replacement for regular care from a board-certified dermatologist, it is important to recognize that, in the context of barriers to care, they are still a helpful way for users to obtain accurate and reliable general information about their disease. Additionally, the comment sections under these videos may provide much-needed validation and support.

Across all video categories, comments discussing users’ personal experiences or giving and receiving advice were significantly more common than comments consisting of critiques, jokes, or off-topic messages (Figure 5). In other words, most comments were “productive” as personal experiences and interuser advice can be helpful for both patients and medical professionals to read and share. This is encouraging as it suggests that the discussions prompted by physician-produced content are likely to remain beneficial and unlikely to become negative, hateful, or unproductive.

Additionally, the fact that sharing personal experiences and advice was more prominent than other types of comments suggests that patients with AD may be using comment sections to seek support. Dermatological conditions, including AD, are often highly visible and impact the quality of day-to-day life for patients. For patients with AD, these videos and comment sections may provide emotionally supportive content by reminding them that others have similar experiences (eg, “Thank you for telling me all this information makes me happy to see other people have eczema. I’m not the only one. Thank you.”), allowing them to vent (eg, “The itching is so horrible but the peeling that happens afterwards is very painful.”), or providing them with nonmedical recommendations that can ease the disease’s visibility or impact on quality of life for the patient (eg, “Also with eczema avoid fragrance in your skin products and avoid anything with alcohol it will dry your skin or ask your dermatologist for the dupixent shot which I’m on and has helped significantly life changing”).

The idea that users may be seeking supportive environments is reinforced by the fact that, among “personal experience” comments, negative personal experiences were more commonly shared than positive or neutral experiences (Figure 6). This suggests that people may have been engaging with the video and potentially finding it in the first place via search terms due to frustration or stress about their disease or in an attempt to find validation as they navigated their AD.

Additionally, among secondary comments, recommendations and emotional support comments trended higher than other types of comments (Figure 7). This shows that users were not only sharing their own experiences and frustrations but also responding to and relating to each other in a transient way. For instance, in response to one comment saying, “I hate having eczema especially when I have it behind my knee it’s so hard to reach,” another user replied, “It really is i feel your pain same here.”

Future studies could further evaluate the quality, reciprocity, and time frame of interactions among commenters to better understand the role that comment sections play in providing a supportive environment. Additionally, it would be helpful to understand whether the same users typically engage with multiple AD-related videos and, thus, form a more connected network.

#### Darker Skin Tones

Notably, we found that only 7.1% (2/28) of the videos had content involving darker skin tones. A previous study found that, when looking specifically for Black skin on TikTok, posts were even less likely to be created by dermatologists compared to general dermatology-related content [22]. Our findings reinforce this content gap and highlight the need for greater inclusivity of darker skin tones in physician-created dermatology content on TikTok. This is especially important when viewing the online spaces created by this content as supportive environments as exclusion of darker skin tones in content may translate to people of color being denied online support and validation.

### Limitations

Our study analyzed TikTok videos retrieved through searches conducted in December 2023. Given the constantly evolving nature of social media, and TikTok in particular, our findings are limited to the period in which the videos were posted (June 2020 to December 2023) and may not reflect content from other time frames. Additionally, studies have critiqued systematic analysis of TikTok content as limited by variability in the content presented to each user based on the app’s algorithm [23]. We attempted to improve replicability by using 3 separate accounts, including 1 newly created account with no established user preferences, to search for AD-related videos. Among the “Top” videos collected by the new account, 60% of the videos (18/30) and 75% (21/28) of the video creators overlapped with the “Top” videos collected by the other 2 accounts, suggesting some similarity among video presentation across users with different personalized algorithms. However, a larger number of varying accounts would need to be studied to confirm this. It is also important to note that the “Top” videos under a search term may not accurately reflect the videos that are most likely to appear in a user’s “For You Page” or personalized feed. The “Top” videos may also not be representative of all AD content on TikTok.

Another limitation is that comment sections represent only a subset of all viewers and may not accurately reflect the general population’s perceptions of these videos. The categorizations in this study were based on interpretations of online comments, and because we did not directly interview individuals, some comments may have been misinterpreted. This is particularly challenging due to the text form nature of comments, which makes tone and intention difficult to judge. Therefore, future research could incorporate qualitative interviews with patients with AD and their loved ones to gain a deeper understanding of how they are affected by these videos and what value they derive from them.

It is also important to note that our methodology excluded all creators whose board certification status was not publicly verifiable, resulting in mostly US-based creators, which may have disproportionately excluded international creators. Additionally, the formula used to calculate the engagement rate may have resulted in inflated engagement rates, particularly for videos with many spam comments or creator replies. Finally, the subset used for interrater reliability only represented 1.7% (20/1149) of the total comment dataset and may constitute a limitation in the reliability of ratings across all samples.

### Conclusions

Our study suggests that videos created by dermatologists on TikTok are generally well received. The comment sections appear to provide transient supportive environments where users connect over shared challenges and exchange personal experiences and recommendations. While previous studies have identified virtual support spaces, they have primarily focused on patient-generated content. Our finding that supportive interactions also occur under physician-produced videos suggests that increasing physician-created content on social media could help expand access to accurate, evidence-based information while maintaining supportive communities for those affected by an emotionally and socially distressing illness such as AD.

## Supplementary material

10.2196/90649 Multimedia Appendix 1 Video metric information ordered by engagement rate.

10.2196/90649 Multimedia Appendix 2 Video categorization ordered by engagement rate.

## Abbreviations

AD: atopic dermatitis
