# Supplementary figures and images for: Comments on Dermatologists’ TikTok Videos on Atopic Dermatitis: Content Analysis
Source: JMIR Dermatol. 2026 Jul 16;9:e90649. doi: 10.2196/90649 (PMC13375941; doi:10.2196/90649)

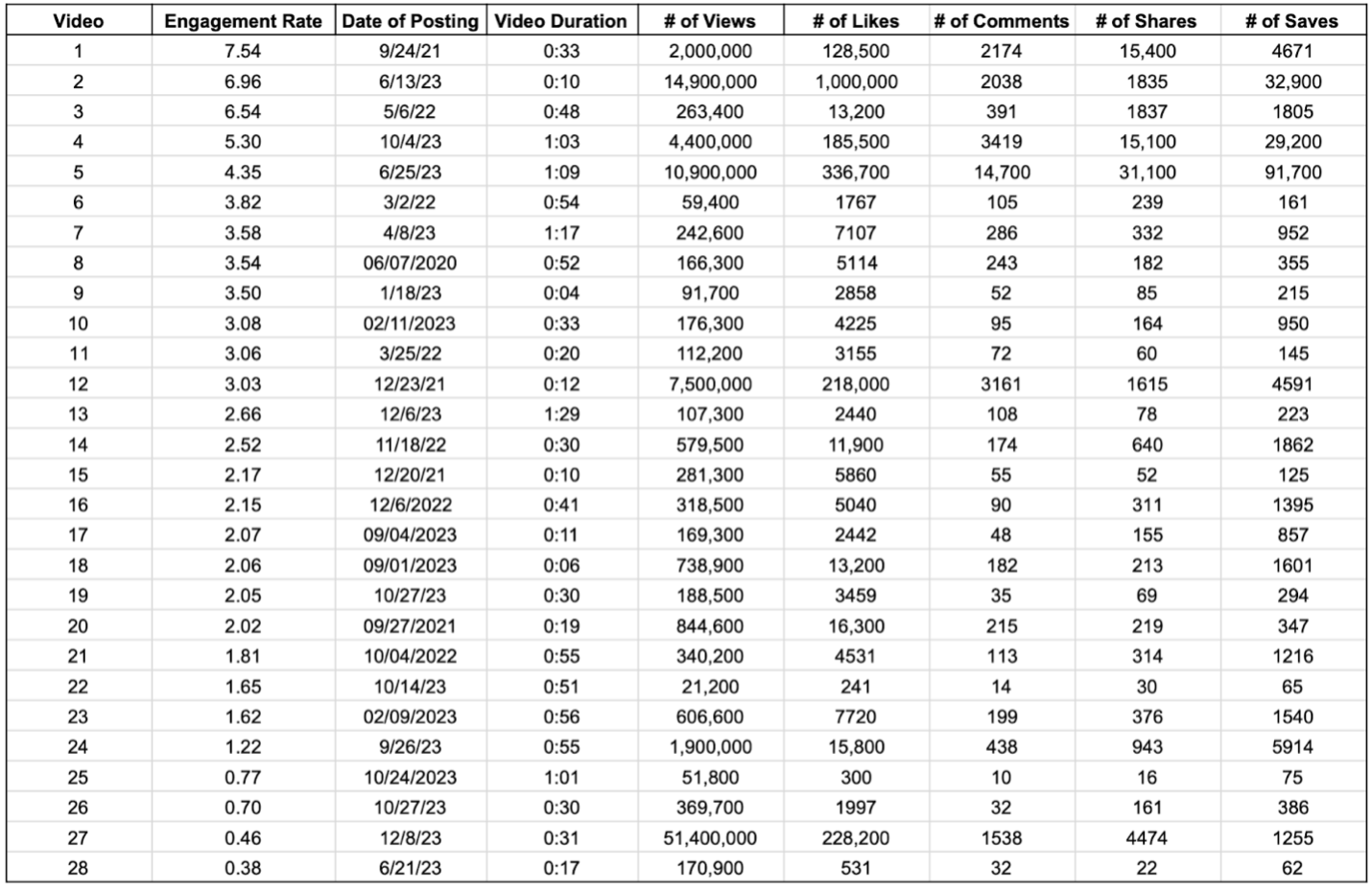

Supplement: Multimedia Appendix 1 [file derma-v9-e90649-s001.png]

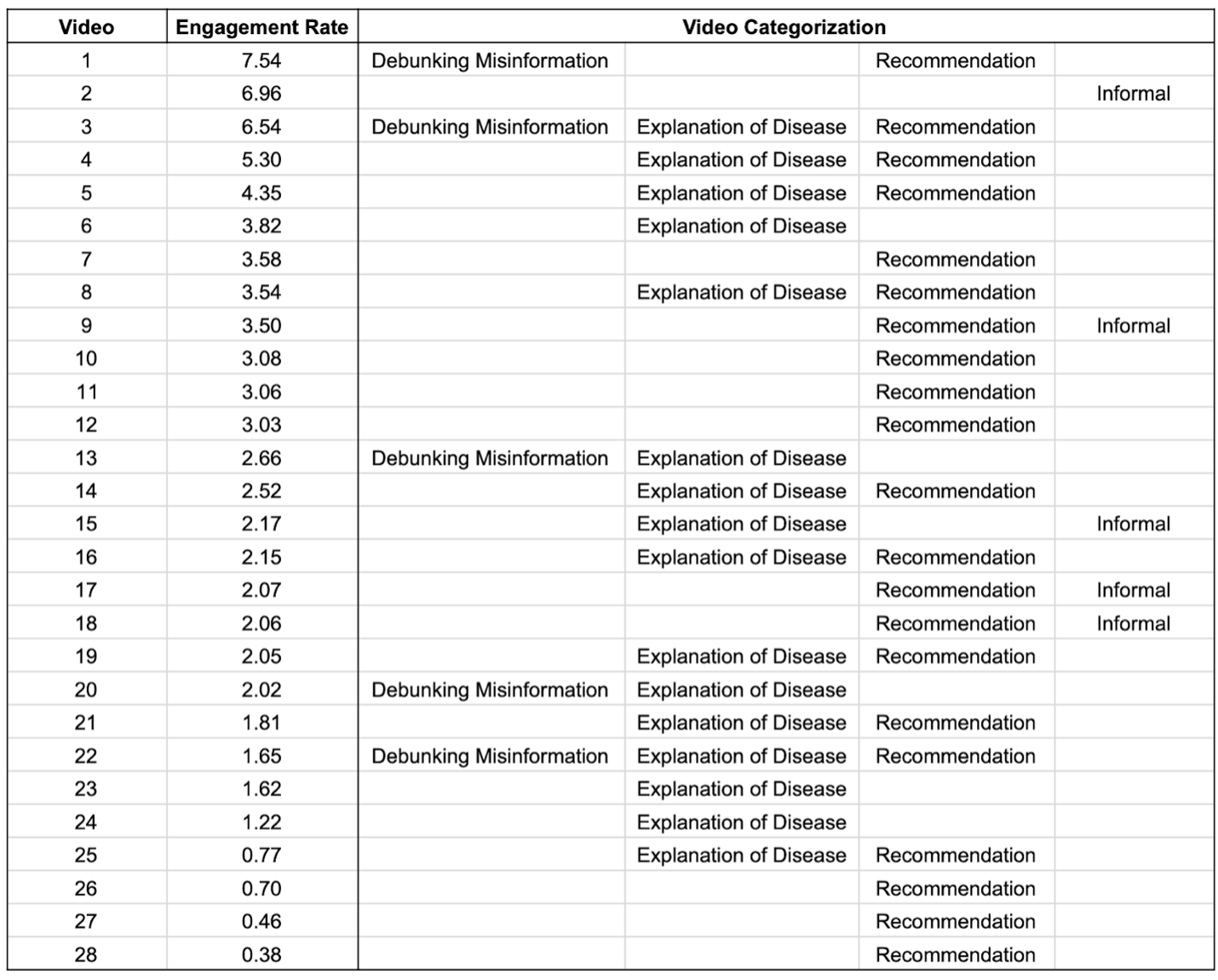

Supplement: Multimedia Appendix 2 [file derma-v9-e90649-s002.png]
